# 615 Evaluation of Topical Off-The-Shelf Therapies to Improve Burn Wound Healing During Prolonged Field Care

**DOI:** 10.1093/jbcr/irac012.243

**Published:** 2022-03-23

**Authors:** David E Varon, Kristo Nuutila, Laura E Cooper, Javier Chapa, Franklin Valdera, Sean E Christy, Robert J Christy, Rodney K Chan, Anders H Carlsson

**Affiliations:** University of Michigan Medical School, San Antonio, Texas; U.S. Army Institute of Surgical Research, San Antonio, Texas; Department of Surgery, University of Maryland Medical Center, San Antonio, Texas; Division of Combat Wound Repair, U.S. Army Institute of Surgical Research, San Antonio, Texas; Department of Surgery, Brooke Army Medical Center, San Antonio, Texas; U.S. Army Institute of Surgical Research, San Antonio, Texas; U.S. Army Institute of Surgical Research, San Antonio, Texas; US Army Institute of Surgical Research, San Antonio, Texas; The Metis Foundation, SAN ANTONIO, Texas

## Abstract

**Introduction:**

Burns are common injuries in the battlefield. Given austere environments, prolonged field care (PFC) is often necessary. Delays in surgical debridement create a risk of infection and deranged healing for burn patients. As such, this study attempts to identify the best commercially available off-the-shelf (OTS) dressings with field-deployable potential.

**Methods:**

Deep-partial thickness burns (1" diameter) were created on the dorsum of 3 anesthetized pigs utilizing a thermocoupled burn device at 100°C for 15s. Non-surgical debridement was done 1-h post-burn creation and either an OTS dressing or standard-of-care (SOC) treatment (Silver Sulfadiazine) was applied to the wound in order to simulate a PFC environment. OTS dressings were randomized and included irradiated sterile human skin allograft (ISHSA), alloplastic absorbable skin substitute (AASS), and synthetic polyurethane dermal matrix (SPDM). Wounds were serially assessed on post-burn days 3, 7, 14, 21, and 28. Assessments were conducted using a combination of photographs, histology, and quantitative bacteriology. Endpoints included burn wound progression, re-epithelialization, wound contraction, scar elevation index (SEI), and colony forming units (CFU).

**Results:**

No statistically significant differences in burn wound progression were seen on days 3 and 7 for the ISHSA or SPDM and the SOC. The differences between the AASS and the SOC were statistically significant on both days (*p≤*0.05). Day 21 re-epithelialization results for the ISHSA, AASS, SPDM and SOC treated wounds were 30%, 85%, 95%, and 78% re-epithelialized, respectively. The difference between the AASS and the SOC was statistically significant (*p≤*0.05). Results showed that by day 28, wound contraction for the ISHSA, AASS, SPDM and SOC treated wounds were 65%, 80%, 82%, and 78%, respectively. No statistically significant differences in wound contraction were seen for any of the OTS dressings and the SOC. SEI showed no statistically significant difference in scar hypertrophy between the OTS dressings and the SOC on day 28. CFU results showed no statistically significant differences between the OTS dressings and the SOC on days 3 and 7.

**Conclusions:**

Three OTS dressings were compared to the SOC for use in the PFC setting. Generally, all the dressings performed well when compared to the SOC in terms of burn wound progression, re-epithelialization, wound contraction, SEI, and CFU. Although significant differences in burn progression and re-epithelialization for burns treated with AASS were seen. In the future, we hope to continue to discover and test various OTS dressings to determine their appropriateness for use in the PFC setting.

